# Social Functioning and Coping Strategies in Romanian and Moldavian Adolescents with Chronic Diseases

**DOI:** 10.1007/s12144-016-9468-5

**Published:** 2016-06-27

**Authors:** Andreea Mihaela Mihalca, Loredana Ruxandra Diaconu-Gherasim, Lacramioara Ionela Butnariu

**Affiliations:** 1 Institute of Psychology, Jagiellonian University in Krakow, 6 Ingardena st., 30-060 Krakow, Poland; 2 Faculty of Psychology and Educational Sciences, Alexandru Ioan Cuza University of Iasi, 3 Toma Cozma st, 700554 Iasi, Romania; 3 Medical Genetics Department, Gr. T. Popa University of Medicine and Pharmacy, 16 University st, 700115 Iasi, Romania

**Keywords:** Adolescence, Chronic disease, Coping strategies, Cross-cultural comparison, Social functioning

## Abstract

The present study aimed to explore the cultural differences in social functioning and coping strategies in chronically ill adolescents. One hundred sixty-eight chronically ill adolescents (45.8 % girls), age 11 to 17 years from Romania (*N* = 78) and Republic of Moldova (*N* = 90) were recruited. Participants filled in self-assessment measures for social functioning problems and coping strategies. Results indicated cross-cultural differences in the studied factors: Moldavian adolescents reported more social functioning problems and higher use of maladaptive coping strategies, while using less adaptive strategies than Romanian counterparts. The associations between social functioning and maladaptive coping strategies were stronger for Romanian than Moldavian adolescents. Further, various coping strategies acted as important predictors for social functioning in the two country samples. Findings suggest that, while the direction of the relation between coping and social functioning in chronically ill adolescents is cultural invariant, the importance played by specific coping strategies in determining social functioning varies by cultural context. Therefore, clinical interventions aimed at improving the social functioning of chronically ill adolescents should take into account the reality of their cultural setting.

## Introduction

Social functioning is defined as the effectiveness of using social competences in social interactions (Cavell 1990). Social functioning could be impaired in the context of chronic diseases, defined as long-term conditions affecting body organs or systems and leaving behind various disabilities (McKenna et al. 1998), by restrictions associated with the medical treatment, hospitalization or disability (La Greca 1990). In this specific context, social functioning could serve as an indicator for parents and health care professionals of possible difficulties with disease management, adherence to treatment or adjustment (Adams et al. 2002; Thompson and Gustafson 1996).

Empirical reviews on chronically ill children’s social functioning suggest that evidence is mixed. Some studies have found no effect of chronic diseases on social functioning (see Spirito et al. 1991 for review), while others found a negative but small decrease in social functioning of chronically ill children compared to healthy ones (see Martinez et al. 2011 for review). An agreement exists only regarding the poor social functioning in children with neurological conditions compared to healthy peers consequent to associated cognitive and physical disabilities which negatively affect social information processing and increase social stigma (see Yeates et al. 2007; La Greca et al. 2002; Martinez et al. 2011 for reviews). Regarding non-neurological conditions, inconsistent findings were reported. Some studies found no decrease in social functioning, not even in conditions which limit participation in social life, such as juvenile rheumatoid arthritis or chronic renal failure (Feldmann et al. 2005; Hooper et al. 2009; Huygen et al. 2000; Reiter-Purtill et al. 2000). Other studies identified a decrease in social functioning in the presence of chronic diseases, such as asthma or chronic renal failure (Blackman and Conaway 2013; Gerson et al. 2010; Varni et al. 2007). These mixed results suggest that the relation between chronic disease and social functioning may be moderated by disease-related characteristics and individual or cultural factors (see Martinez et al. 2011 for review; Livneh and Wilson 2003; Meijer et al. 2002).

Among the individual factors, coping strategies are recognized as important mediators between disease-related stress and individual’s psychological adjustment (Lazarus and Folkman 1984; Lazarus 2000). Nevertheless, few studies have explored the relation between coping and social functioning in chronically ill adolescents (Greene et al. 2006; Meijer et al. 2002). Further, because the majority of studies on both coping (Kuo 2010) and social functioning (Blackman and Conaway 2013) were performed in Western English-speaking countries, the generalizability of the results on non-Western cultures is hard to sustain (Henrich et al. 2010). Therefore, in an effort to increase the understanding of social functioning in chronically ill adolescents in relation to coping strategies in non-Western cultures, this present study aimed to explore the relation between social functioning and coping strategies in adolescents with chronic diseases living in different cultural contexts from Eastern Europe.

## Social Functioning and Coping Strategies

Coping strategies are defined as individuals’ cognitive or behavioral efforts used to deal with stressful situations (Lazarus and Folkman 1984; Lazarus 2000). Coping strategies are most commonly classified into problem-focused, aimed at changing the stressful situation, versus emotion-focused, aimed at changing the emotions experienced during the stressful situation (Lazarus and Folkman 1984; Lazarus 2000). Engagement coping, oriented on both the source of stress and associated emotional response, versus disengagement coping, oriented on avoiding the stressors and emotional response (Ebata and Moos 1991), is another very commonly used classification. Based on the similarity in the conceptualization between emotion-focused coping and emotion regulation – as both concepts refer to the management of emotions experienced when confronted with negative situations (Lazarus and Folkman 1984; Thompson 1994) – it was suggested to combine the research from coping and emotion regulation fields (Garnefski et al. 2001; Losoya et al. 1998). In the present study we followed this recommendation and considered both emotion-focused coping and emotion regulation as a single concept comprising those strategies aimed at regulating negative emotions. In other words, we considered empirical evidence from both emotion coping and emotion regulation fields in relation to social functioning.

Generally, previous studies have found consistent positive associations between both engagement and problem-focused coping, such as cognitive restructuring and social competences in children and adolescents dealing with various life stressors (see Compas et al. 2001 for review). Contradictory results were found regarding disengagement and emotion-focused coping: some studies showed positive associations, while others reported negative associations between these coping strategies and children’s social functioning (see Compas et al. 2001 for review). Nevertheless, the studies investigating social functioning in the field of emotion regulation revealed positive associations between children’s social functioning and the use of emotion regulation strategies (Eisenberg et al. 2001; Spinrad et al. 2006). These contradictory results highlight the need of putting coping in context; emotion-focused strategies seem more beneficial than problem-focused ones when adapting to uncontrollable stressors, such as chronic diseases (Austenfeld and Stanton 2004; Weisz et al. 1994).

Nevertheless, the relation between emotion-focused strategies and social functioning was scarcely analyzed during adolescence and particularly in the context of chronic diseases (Compas et al. 2001). Instead, this relation was extensively explored in healthy children younger than six (Eisenberg et al. 2000; Losoya et al. 1998). The few existing studies conducted on chronically ill adolescents have provided inconsistent results. Some studies found no association between either avoidance or problem-focused strategies and social withdrawal in chronically ill children (Greene et al. 2006). Other studies found positive associations between problem-focused strategies and social competences in chronically ill children, while avoidance coping was not related to their social functioning (Meijer et al. 2002). Different explanations were given for these contradictory findings. Some researchers emphasized that the strength of the relation between coping strategies and social functioning is dependent on the degree of diseases related-stress; the relation being stronger in high stressful situations (Greene et al. 2006). Other researchers suggested that the relation vary with respect to contextual factors, including cultural groups (Martinez et al. 2011).

## Social Functioning, Coping Strategies and Culture

Cultural values and norms may shape the appraisal of stressor and regulate individuals’ social behavior, including social functioning and coping responses (Lazarus and Folkman 1984; Matsumoto et al. 2008; Schwartz 2006). For example, in African countries, adolescents with chronic conditions have impaired social functioning because social norms reduce social interactions and increase stigmatization of individuals with chronic conditions (Povlsen and Ringsberg 2008). Few previous studies have been performed on children samples from non-Western countries (Blackman and Conaway 2013; Kuo 2010). Among the non-Western cultures, the East-European culture is least represented (Kuo 2010).

It is important to note that more research in East-European countries is needed considering that the literature reveals important cultural differences even between Western and Eastern Europe (Inglehart and Baker 2000; Schwartz 2006), which impedes the generalizability of the findings. For example, Eastern European countries are characterized by high embeddedness (emphasis on traditional order, role expectation and social interdependence) and hierarchy (emphasis on social differentiation of roles, power and resources) while the opposite is observed in Western European countries which are high on autonomy (emphasis in self-determination and independent decision-making) and egalitarianism (emphasis on equality in chance and resources for all people). Moreover, the dissimilarity in the socio-economic development is translated into a higher availability of better medical treatment in Western industrialized countries than in Eastern developing ones (Pinquart and Shen 2011). Consequently, severity of disease in Western countries is lower compared to Eastern ones (Gutiérrez-Suárez et al. 2007).

Additionally, little research has been conducted in the field of social functioning and coping. This is of particular importance considering that previous findings indicated differences in social functioning between Western and non-Western cultures. For example, adolescents from South Africa reported more social functioning problems than adolescents from Canada or European countries (Wildhaber et al. 2012). Cross-cultural differences in the preference of using coping strategies have also been identified. Specifically, older adolescents from Asian countries used more emotion-focused coping, while teens from Western countries preferred more problem-focused strategies when dealing with stressful events (Essau and Trommsdorff 1996; Oláh 1995; Sinha et al. 2000). Moreover, previous literature has indicated that the relation between coping and adjustment to illness varies by culture. As such, the use of emotion-focused coping was associated with a decrease in physical symptoms in young adults from Western cultures, but with an increase in patients from Asian cultures (Essau and Trommsdorff 1996). To our knowledge, no previous cross-cultural study has explored whether there are differences in the relation between coping and social functioning in chronically ill adolescents from Eastern European countries based on their cultural group.

## The Present Study

In this research we first aimed to explore whether there are cultural differences in social functioning and coping strategies among chronically ill adolescents from two Eastern European countries, namely Romania and the Republic of Moldova.

Despite the similarities between the two countries, which arise from their shared history (the Republic of Moldova was once a part of Romania) and shared language (Romanian is the official langue in both countries), we expected to find differences in chronically ill adolescents’ social functioning and coping strategies consequent to the disparities in the socio-economic development and cultural values. Specifically, the lower human development and health indexes of the Republic of Moldova compared to Romania (United Nations Development Programme (UNDP) 2014) reflect differences in the organization of the health care system, the budget available for the medical system, and patients’ health education and access to medical services. These differences may lead to various progression of the chronic disease. In other words, Moldavian adolescents may experience more severe chronic disease compared to Romanian ones. Considering that disease severity is a stressful event associated to adjustment problems (MacLean et al. 1992) and the activation of coping strategies (Lazarus and Folkman 1984; Lazarus 2000), we expected Moldavian adolescents to report lower social functioning while using more coping strategies than their Romanian counterparts.

This hypothesis was also based on the differences in the cultural values. Specifically, the Republic of Moldova is characterized by extreme scores on the survival dimension, while Romania, although also being on the survival pole, is not as extreme (Inglehart and Baker 2000). The survival dimension comprises, among others, low levels of health, subjective well-being and interpersonal trust, sustaining therefore our hypothesis regarding the lower social functioning and increased need of using coping strategies in chronically ill Moldavian adolescents compared to their Romanian peers.

The second aim of the study was to explore whether there are cultural differences in the relation between coping strategies and social functioning of chronically ill adolescents originating from Eastern European countries. Based on the previous literature emphasizing a stronger relation between coping and social functioning in the presence of high levels of stress (Greene et al. 2006), we expected to find stronger associations in Moldavian than in Romanian adolescents.

In this study, we focused on adolescents diagnosed with chronic renal failure (CRF), juvenile rheumatoid arthritis (JRA) and asthma. We recruited children with CRF and JRA based on previous findings on adult samples which found a significant decrease in social functioning of patients with these diseases (Sprangers et al. 2000). Further, we selected adolescents with asthma considering the high prevalence of this disease (Sennhauser et al. 2005). According to the results from previous comparative studies including children with non-neurological conditions, no differences in social functioning were expected based on the type of disease (Adams et al. 2002; Meijer et al. 2000; Siefert et al. 1992).

## Method

### Participants and Procedure

The sample included 168 adolescents (48.5 % girls) aged between 11 and 17 years old (*M* = 13.86, *SD* = 1.86), from Romania (*N* = 78; 52.6 % girls; age *M* = 13.99, *SD* = 2.03) and the Republic of Moldova (*N* = 90; 40 % girls; age *M* = 13.76, *SD* = 1.64). The participants were recruited from pediatric hospitals on a voluntary basis. Prior to their participation, parents and adolescents aged 16 or above signed in an informed consent. The research procedure was approved by the ethical committee of the hospitals where the participants were recruited. Adolescents filled in the self-assessment measures during their periodical visit or during their hospitalization. From the total sample, 35.7 % participants had been diagnosed with asthma, 35.7 % with CRF and 28.6 % with JRA. The Romanian sample included 30 adolescents diagnosed with asthma (12 girls; age *M* = 13.47, *SD* = 1.75), 30 with CRF (14 girls; age *M* = 14.03, *SD* = 2.23) and 18 with JRA (15 girls; age *M* = 14.78, *SD* = 1.95). The Moldavian sample included 30 adolescents diagnosed with asthma (14 girls; age *M* = 13.53, *SD* = 1.65), 30 with CRF (14 girls; age *M* = 13.77, *SD* = 1.52) and 30 with JRA (8 girls; age *M* = 13.97, *SD* = 1.75). There were no significant differences in the participants’ age between cultural groups by type of disease, *F* = 1.39, *p* > .05. Further, there were no significant differences in gender distribution in disease groups, χ^2^s < 1.66, *p*s > .05, country groups, χ^2^s < 3.36, *p*s > .05 or country x type of disease groups, χ^2^s < 1.12, *p*s > .05, except for the JRA. In the Moldavian JRA group there were more boys than girls, while in the Romanian JRA group there were more girls than boys, χ^2^s < 8.00, *p*s < .001.

### Instruments

The adolescents’ social functioning was assessed with an adapted version of the *Living with Chronic Illness Scale* – *Youth form* (LCI-y; Adams et al. 2002). The scale is composed by 29 items requiring three types of answers, referring to the presence of social functioning problems, the association between social problems and chronic disease as well as the distress associated with those problems. In this study only the information referring to the presence of social functioning problems was used. Adolescents rated the presence of social problems on a dichotomous scale (*0* = yes and *1* = no). The total score was computed by summing the items scores; high scores represented more social functioning problems. Previous studies have shown that the scale has good psychometric properties in that it correlates with measures of adolescents’ behavioral, emotional and social problems as well as with measures for self-perceived competences (Adams et al. 2002). The internal consistency was .79 for the entire sample, .82 for the Romanian sample, respectively .74 for the Moldavian sample.

The participants’ emotion-focused coping strategies were measured with the *Cognitive Emotion Regulation Questionnaire - Kids* (CERQ-k; Garnefski et al. 2007). The 36-item questionnaire consists of nine subcales (4 items per subscale) measuring five maladaptive (Self-blame, Acceptance, Rumination, Catastrophizing and Other-blame) and four adaptive (Putting into Perspective, Planning, Positive Reappraisal and Positive Refocusing) coping strategies. The participants answered each item on a five-point Likert scale (*1* = almost never to *5* = almost always). For each subscale, total scores were computed by summing the items’ scores; higher scores indicating a higher frequency of using a specific coping strategy when dealing with disease-related stress. The instrument has demonstrated good internal reliability and consistent correlations with various emotional outcomes, such as depression, fear or worries (Garnefski et al. 2009). The internal consistency was satisfactory for the majority of subscales, the Alphas coefficients ranging from .59 to .93 for Romanian sample, from .53 to .63 for Moldavian sample, and from .54 to .78 for the entire sample. Due to low internal consistencies (α < .50), the Acceptance, Positive Reappraisal and Putting into Perspective subscales were not considered.

The instruments were translated from English into Romanian language using the forward-backward translation design (Hambleton et al. 1999). Minor corrections to the translations were made based on the back-translation process. The Romanian version of the instruments was pretested for use in the Republic of Moldova where Romanian is the official language.

## Results

We conducted preliminary analyses to investigate whether the adolescents’ age, gender and type of disease were related to social functioning. We also analyzed the associations among the main study variables. Then, we examined whether there were cultural group differences in the use of coping strategies, the number of social functioning problems, and the relations between the coping strategies and social functioning problems. Finally, we did additional analyses to test whether cultural group moderated the associations between coping strategies and social functioning. For each analysis we discuss first the results based on the entire sample, followed by the results based on each cultural group.

### Preliminary Analyses

The multivariate factorial analysis of covariance with all variables revealed no significant interactions between the cultural group and the participants’ gender or type of disease on the study variables. Moreover, the participants’ age had no significant correlation with the LCI-y or CERQ-k subscale scores, (*r*s < .12, *p*s > .05). Consequently, demographic variables were not included in subsequent analyses.

Zero-order correlations among adolescents’ coping strategies ranged from .14 to .70, all *p*s < .01, based on the entire sample, except for associations between Positive Refocusing and both Catastrophizing and Other-blame (see Table 1).

**Table 1:** Descriptive statistics and correlations between the analysed variables for the entire sample

	*1*	*2*	*3*	*4*	*5*	*6*	*7*
1. Self-blame	_						
2. Rumination	.54***	_					
3. Catastrophizing	.58**	.70***	_				
4. Other-blame	.48**	.42***	. 42***	_			
5. Planning	.19*	.41***	.23***	.14	_		
6. Positive refocusing	−.15*	.14	.00	−.07	.40***	_	
7. LCI-y	.28***	.30***	−.37***	.39***	−.11	−.16*	_
Mean	9.08	11.50	10.64	8.06	13.49	13.58	10.79
SD	4.06	3.97	3.82	3.62	3.49	3.34	5.23

*N* = 168; LCI-y - Living with Chronic Illness Scale – youth form

* *p* < .05; ** *p* < .01; *** *p* < .001

As presented in Table 2, similar results were observed for each cultural group. For the Romanian sample, *r*s ranged from .10 to .70, all *p*s < .05, except for the associations between Positive Refocusing and both Catastrophizing and Other-blame (*rs* < .09, all *p*s > .05). For the Moldavian sample, *r*s ranged from .12 to .58, all *p*s < .05, except the associations between Positive Refocusing and both Self-blame and Other-blame (*rs* < .00, all *ps* > .05).

**Table 2:** Descriptive statistics and correlations between the analysed variables for each cultural group

	*1*	*2*	*3*	*4*	*5*	*6*	*7*	*Mean*	*SD*
1. Self-blame	_	.20^†^	.43***	.52***	.12	.01	.10	10.82	3.55
2. Rumination	.63***	_	.58***	.30***	.37***	.44***	.02	12.82	2.73
3. Catastrophizing	.53***	.70***	_	50***	31***	.36***	.07	12.07	3.01
4. Other-blame	.14	.32**	.11	_	.21*	.00	.18^†^	9.64	3.20
5. Planning	.29**	.47***	.22^†^	.11	_	.49***	−.24*	13.50	2.96
6. Positive refocusing	−.16	. 09	−.13	.02	.35**	_	−.04	13.03	3.08
7. LCI-y	.23*	.34**	.45***	.41***	−.02	−.19	_	12.37	4.87
Mean	7.06	9.97	8.99	6.23	13.49	14.21	8.97		
SD	3.68	4.60	4.01	3.22	4.03	3.54	5.05		

LCI-y - Living with Chronic Illness Scale – youth form; Lower left - zero-order associations for Romanian sample (*N* = 78); Upper right - zero-order associations for Moldavian sample (*N* = 80)

†*p* < .10; *p < .05; **p < .001; ***p < .001

### Cross-Cultural Differences in Adolescents’ Social Functioning and Coping Strategies

There was a main effect of cultural group on adolescents’ social functioning problems, *t* (166) = 4.42, *p* < .01, *d* = .69. Specifically, Moldavian adolescents reported higher scores on the LCI-y than their Romanian peers (see Table 2). There was a main effect of cultural group on all the negative coping strategies, namely Self-blame, Rumination, Catastrophizing and Other-blame, *t*s (166) = 6.73, 4.79, 5.57 and 6.88, *d*s = 1.04, .75, .87 and 1.08, all *p*s < .05. There was also a significant main effect of the cultural group on the use of one positive coping strategy, namely Positive Refocusing, *t* (166) = 2.29, *p* < .05, *d* = .36. As shown in Table 2, Moldavian adolescents reported significantly higher scores on Self-blame, Rumination, Catastrophizing, and Other-blame, but lower scores on Positive Refocusing compared to Romanian counterparts.

### Cultural Differences in the Relation between Social Functioning and Coping Strategies

The associations between adolescents’ coping strategies and social functioning problems on the entire sample combined (see Table 1) indicated that adolescents who used more maladaptive strategies, such as Self-blame, Rumination, Catastrophizing, and Other-blame (*r*s = .28, .30, .37, and .39, respectively, all *p*s < .001), but less adaptive strategies, such as Positive Refocusing (*r* = −.16, *p* < .05) reported more social functioning problems. When the pattern of associations was examined for each cultural group (see Table 2), the results were replicated only in the Romanian sample. For the Moldavian sample, all associations were non-significant (*r*s < .17, *p*s > .05), except the link between Planning and social functioning problems (*r* = −.24, *p* < .01). Nevertheless, significant differences were found across cultural groups only for the relations between the LCI-y scores and both Rumination (*Z* = 2.18, *p* < .05) and Catastrophizing (*Z* = 2.63, *p* < .05). All these findings showed that the relations between these coping strategies and social functioning were stronger in Romanian than in the Moldavian sample.

**Fig. 1:**
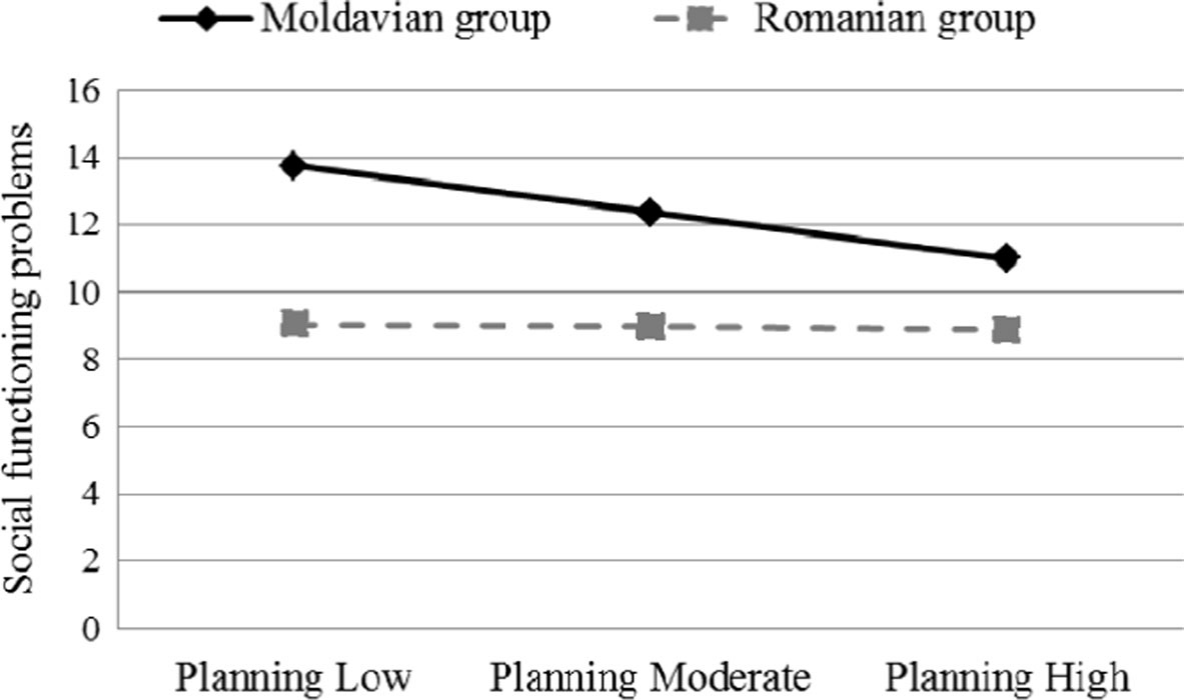
The moderating role of culture in the relation between planning and social functioning

**Fig. 2:**
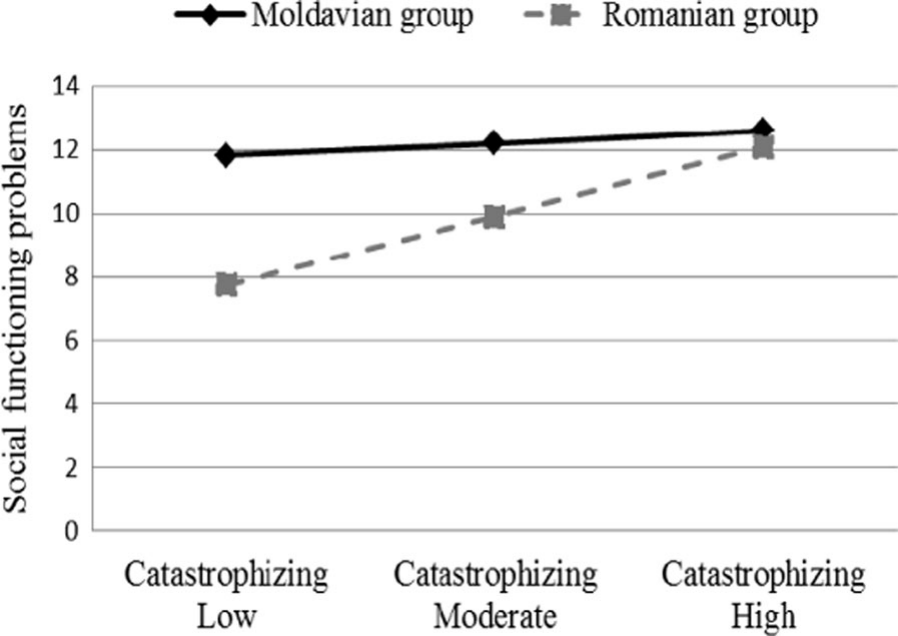
The moderating role of culture in the relation between catastrophizing and social functioning

Additionally, to test whether the cultural group moderated the relation between adolescents’ coping strategies and their social functioning, we performed multiple hierarchical regression analyses with social functioning problems as the dependent variable (Cohen et al. 2013). The coping strategies were entered in Step 1, the cultural group was entered in Step 2, and the interactions between coping strategies and cultural group were entered in Step 3. The results indicated that the coping strategies collectively explained 23.5 % of the variance in social functioning (Δ*R* ^2^ = .23, *F* (6, 161) = 9.54, *p* < .01). As seen in Table 3, the LCI-y scores were predicted negatively by Planning (β = −.22, *p* = .007) and positively by Catastrophizing and Other-blame (β = .21 and .28 respectively, all *p*s < .05). The cultural group did not add significantly to the variance explained (Δ*R* ^2^ = .004, *F* (1, 160) = .89, *p* > .05) and was not a significant predictor of social functioning problems (β = −.08, *p* > .05). Further, the Planning x cultural group as well as Catastrophizing x cultural group interactions (βs = .25 and .31, all *p*s < .055) were marginally significant predictors for the LCI-y scores. The interaction terms explained a marginally significant additional variance in social functioning (Δ*R* ^2^ = .06, *F* (6, 154) = 2.14, *p* = .051). The nature of these interactions is illustrated in Figs. 1 and 2.

**Table 3:** Hierarchical multiple regression analyses to predict adolescents’ social functioning problems

*Variables*	*ΔR* ^*2*^	*B*	*SE*	*β*
*Step 1*	.23***			
Self-blame		−.12	.47	−.02
Rumination		.78	.55	.15
Catastrophizing		1.10	.53	.21*
Other-blame		1.45	.42	.28**
Planning		−1.16	.42	−.22**
Positive Refocusing		−.42	.40	−.08
*Step 2*	.004			
Cultural group		−.82	.87	−.08
*Step 3*	.06*			
Cultural Group x Self-blame		−.35	1.01	−.04
Cultural Group x Rumination		−.14	1.22	−.02
Cultural Group x Catastrophizing		2.20	1.13	. 31†
Cultural Group x Other-blame		.76	.93	.10
Cultural Group x Planning		1.66	.86	. 25†
Cultural Group x Positive Refocusing		−1.27	.86	−.18

*N* = 168; *p < .05, **p < .01, ***p < .001, †*p* < .10

## Discussion

This present study investigated whether there are culture-based differences in social functioning and coping strategies of chronically ill adolescents. Specifically, our first aim was to examine whether social functioning and coping strategies differ as a function of their culture, by comparing chronically ill adolescents from Romania and the Republic of Moldova. The second goal was to explore whether the relation between coping strategies and social functioning varies by cultural context.

Consistent with our first hypothesis the results revealed cultural differences in social functioning and coping strategies, indicating that Moldavian chronically ill adolescents reported more social functioning problems than their Romanian counterparts. Also, in dealing with a chronic disease the Moldavian adolescents reported a higher use of coping strategies such as self-blame, rumination, catastrophizing and other-blame, while reporting a lower use of positive refocusing than their Romanian peers. In other words, Moldavian adolescents used more maladaptive coping strategies, but less adaptive ones than their Romanian counterparts. These results can be explained by the socio-economic and cultural differences between these two countries. Namely, the extreme position of the Republic of Moldova on the cultural dimension of survival could be translated into low levels of health, subjective well-being and interpersonal trust (Inglehart and Baker 2000). Further, the low socio-economic development of the Republic of Moldova compared to Romania, as indicated by the differences in the human development index and health index (UNDP 2014), could result into higher severity of the disease and worse health status in Moldavian adolescents than in their Romanian peers. Disease severity is known to be positively associated with adjustment problems (MacLean et al. 1992), which in turn activates a higher use of coping strategies (Greene et al. 2006).

The results also sustained our second hypothesis, indicating cultural differences in the relation between coping and social functioning in chronically ill adolescents. In line with previous studies our findings revealed a small to moderate relation between coping strategies and social functioning (Clarke 2006; Compas et al. 2001). Further, consistent with previous findings from the field of emotional adjustment (Garnefski et al. 2003; Legerstee et al. 2010), catastrophizing, rumination and other-blame were risk factors, while planning was a protective factor for social functioning in both Romanian and Moldavian adolescents. Nevertheless, contrary to previous findings indicating that both catastrophizing and rumination are the most important predictors for emotional adjustment (Garnefski et al. 2003; Legerstee et al. 2010), our results revealed that other-blame was the most important predictor for social functioning problems in chronically ill adolescents. These findings indicate that the relative importance of coping strategies depends on the nature of adjustment outcome.

However, the strength between social functioning and two coping strategies – rumination and catastrophizing – varied based on cultural groups. Specifically, the associations between these coping strategies and social functioning problems were stronger for Romanian adolescents than for their Moldavian counterparts. Further, the regression analysis showed that the relations of both catastrophizing and planning with social functioning were moderated by cultural contexts. Catastrophizing was the most important maladaptive strategy in the Romanian sample, while planning was the most important adaptive strategy for Moldavian adolescents. Specifically, Romanian chronically ill adolescents who use more catastrophizing in dealing with their chronic disease reported more social functioning problems than those using less this strategy. Moldavian adolescents reported similar social functioning regardless of the frequency of using catastrophizing. Instead, chronically ill Moldavian adolescents who use less planning reported more social functioning problems than those who use this coping strategy more frequently, whereas Romanian adolescents reported similar social functioning problems regardless of the frequency of using this strategy.

The lack of previous cross-cultural studies makes it difficult to explain why some strategies are more important than others. It is possible that in Moldavian adolescents the use of planning is associated with higher perceived control over their disease, which would facilitate better adjustment to the disease. On the other side, Romanian adolescents may consider that they do all they can when it comes to planning, but without impact on their disease. These current findings suggest that psychological intervention in decreasing the use of catastrophizing when coping with a chronic disease could lead to a decrease in social functioning problems only in Romanian adolescents, while not having any impact on the social functioning of Moldavian adolescents. Instead, chronically ill Moldavian adolescents would benefit more from an intervention aimed at increasing the use of planning.

The present findings should be interpreted considering the study’s limits. Firstly, we did not control for other disease related factors which may explain the relation between coping and social functioning, such as disease severity or type of treatment. Therefore, the samples were not matched on these characteristics. Instead, the difference between the two selected countries in disease severity was hypothesized based on socio-economic factors and cultural values. Nevertheless, the results are in the expected direction according to the hypothesized differences in disease severity. Future cross-cultural studies on social functioning controlling for disease severity using objective measures are needed. Secondly, the correlational design of this study does not provide evidence for the causal associations between coping and social functioning. Longitudinal studies could provide a better understanding of the dynamic relation between these factors. Thirdly, we used only self-report measures, thus providing only the adolescents’ point of view regarding their social functioning problems. A clearer picture could be obtained following the inclusion of multiple informants, such as parents, teachers or peers.

This current study contributes to the existing body of evidence concerning the role of coping in chronically ill adolescents’ social functioning in some important ways. Firstly, while the majority of studies have previously examined how coping strategies are associated to children’s psychological adjustment, their associations with social functioning in chronically ill adolescents have been overlooked (Greene et al. 2006; Meijer et al. 2002). Secondly, little research has analyzed coping strategies and social functioning across non-Western cultures (Blackman and Conaway 2013; Kuo 2010). Thus, the present research increases the generalizability of coping and social functioning models on other non-Western cultures, such as the East-European one. Thirdly, this study is among the first to show cultural differences in the importance played by coping strategies in the social functioning of chronically ill adolescents.

To sum up, our results indicate that while the direction of the relation between coping and social functioning in chronically ill adolescents is cultural invariant and similar with the one identified in Western studies, the importance played by specific coping strategies in determining social functioning varies by cultural context. Clinical interventions aimed at helping chronically ill adolescents cope with disease and improve social functioning should be developed based on the results of studies which take into account the reality of their cultural setting.
